# The MetaCyc database of metabolic pathways and enzymes and the BioCyc collection of Pathway/Genome Databases

**DOI:** 10.1093/nar/gkt1103

**Published:** 2013-11-12

**Authors:** Ron Caspi, Tomer Altman, Richard Billington, Kate Dreher, Hartmut Foerster, Carol A. Fulcher, Timothy A. Holland, Ingrid M. Keseler, Anamika Kothari, Aya Kubo, Markus Krummenacker, Mario Latendresse, Lukas A. Mueller, Quang Ong, Suzanne Paley, Pallavi Subhraveti, Daniel S. Weaver, Deepika Weerasinghe, Peifen Zhang, Peter D. Karp

**Affiliations:** ^1^SRI International, 333 Ravenswood, Menlo Park, CA 94025, USA, ^2^Carnegie Institution, Department of Plant Biology, 260 Panama Street, Stanford, CA 94305, USA and ^3^Boyce Thompson Institute for Plant Research, Tower Road, Ithaca, New York 14853 USA

## Abstract

The MetaCyc database (MetaCyc.org) is a comprehensive and freely accessible database describing metabolic pathways and enzymes from all domains of life. MetaCyc pathways are experimentally determined, mostly small-molecule metabolic pathways and are curated from the primary scientific literature. MetaCyc contains >2100 pathways derived from >37 000 publications, and is the largest curated collection of metabolic pathways currently available. BioCyc (BioCyc.org) is a collection of >3000 organism-specific Pathway/Genome Databases (PGDBs), each containing the full genome and predicted metabolic network of one organism, including metabolites, enzymes, reactions, metabolic pathways, predicted operons, transport systems and pathway-hole fillers. Additions to BioCyc over the past 2 years include YeastCyc, a PGDB for *Saccharomyces cerevisiae*, and 891 new genomes from the Human Microbiome Project. The BioCyc Web site offers a variety of tools for querying and analysis of PGDBs, including Omics Viewers and tools for comparative analysis. New developments include atom mappings in reactions, a new representation of glycan degradation pathways, improved compound structure display, better coverage of enzyme kinetic data, enhancements of the Web Groups functionality, improvements to the Omics viewers, a new representation of the Enzyme Commission system and, for the desktop version of the software, the ability to save display states.

## INTRODUCTION

MetaCyc (MetaCyc.org) is a highly curated nonredundant reference database of small-molecule metabolism. It contains metabolic pathway and enzyme data that have been experimentally validated and reported in the scientific literature (1). Owing to its exclusively experimentally determined pathways and enzymes, intensive curation and tight integration of data and references, MetaCyc is a uniquely valuable resource for various fields including biochemistry, enzymology, genome and metagenome analysis and metabolic engineering. The metabolic pathways and enzymes in MetaCyc are derived from all domains of life.

In conjunction with its role as a general reference on metabolism, MetaCyc can be used as a reference database for the PathoLogic component of the Pathway Tools software (2) to computationally predict the metabolic network of any organism that has a sequenced and annotated genome (3). During this automated process, a predicted metabolic network is created in the form of a Pathway/Genome Database (PGDB). In addition to the automated creation of PGDBs, Pathway Tools has editing capabilities that enable scientists to improve and update these computationally generated PGDBs by manual curation. MetaCyc has been used by SRI International (SRI) to create >3000 PGDBs (as of October 2013), which are available through the BioCyc (BioCyc.org) Web site. Interested scientists may adopt any of these PGDBs through the BioCyc Web site for further curation (biocyc.org/intro.shtml#adoption).

MetaCyc is also used by other scientists to create additional PGDBs, many of which are available to the public via the scientists’ own Web sites. Together with BioCyc, these PGDBs form the *MetaCyc family of databases* (4).

More than 250 groups have used Pathway Tools and MetaCyc to create PGDBs for their organisms of interest, including important model organisms such as *Saccharomyces cerevisiae* (5), *Arabidopsis thaliana* (6), *Oryza sativa* (7), *Mus musculus* (8), *Bos taurus* (9), *Medicago truncatula* (10), *Populus trichocarpa* (11), *Dictyostelium discoideum* (12), *Leishmania major* (13), *Chlamydomonas reinhardtii* (14), several *Solanaceae* species (15), bioenergy-related organisms (BeoCyc) and many pathogenic organisms (16) (see http://biocyc.org/otherpgdbs.shtml for a more complete list). Examples of organisms that were studied during the previous 2 years using Pathway Tools include archaea (17,18), bacteria (19–49), fungi (50–54), a diatom (55), plants (56–59) and lower eukaryotes (60,61). In addition, Pathway Tools is used to analyze data from the Human Microbiome Project (62–66) and other metagenomic data sets (27,67,68).

A web server included in Pathway Tools enables the publishing of PGDBs through either the Internet or an internal network, and the Navigator component of Pathway Tools allows the browsing and analyzing of PGDBs, either locally or over the Internet. A detailed description of Pathway Tools can be found in (2).

PGDBs generated by Pathway Tools and MetaCyc are an excellent platform for the integration of genome information with many other types of data comprising metabolism, regulation and genetics. They provide powerful tools for analyzing omics data sets from experiments related to gene transcription, metabolomics, proteomics, ChIP-chip analysis and other resources. During the past 2 years, we again significantly expanded the data content of MetaCyc and BioCyc, and added supporting enhancements to the Pathway Tools software and BioCyc Web site, as described in the following sections.

## METACYC ENHANCEMENTS

### Expansion of MetaCyc

All pathways in MetaCyc are curated from the experimental literature. Since the last *Nucleic Acids Research* publication (2 years ago) (1), we added 384 new base pathways (pathways comprised of reactions only, where no portion of the pathway is designated as a subpathway) and 33 superpathways (pathways composed of at least one base pathway plus additional reactions or pathways), and updated 154 existing pathways, for 538 new and revised pathways. The total number of base pathways grew by 17%, from 1790 (version 15.5) to 2097 (version 17.5) (the total increase is <384 pathways because some existing pathways were deleted from the database during this period). A comparison of MetaCyc 16.0 and a kyoto encyclopedia of genes and genomes (KEGG) version downloaded on 27 February 2012 showed that MetaCyc contained significantly more reactions and pathways than did KEGG, although the number of reactions occurring in pathways in the two databases was similar (69).

Along with the increase in pathway number, the number of enzymes, reactions, chemical compounds and citations in the database grew by 20, 19, 11 and 21%, respectively; the number of referenced organisms increased by 18% (currently at 2460). See Table 1 for a list of species with >20 experimentally elucidated pathways in MetaCyc, and Table 2 for the taxonomic distribution of all MetaCyc pathways.

### Atom mapping

A reaction atom mapping describes for each atom of a reactant (excluding hydrogens) its corresponding atom in the product. Implicitly, an atom mapping illustrates which bonds are broken and created during the reaction. Atom mapping information is depicted in PGDB reactions by coloring conserved chemical moieties within a reaction (currently available only in the Firefox and Chrome browsers). In addition, if the user hovers the mouse over an atom in a reactant, the corresponding atom is highlighted in the product (again, only in the Firefox and Chrome browsers).

The atom-mapping data of each reaction can also be downloaded from the MetaCyc Web site after the reaction page is displayed by selecting the ‘Download atom mapping(s) for this reaction’ command from the right side bar. All atom mappings for MetaCyc are stored in one flat file, atom-mappings.dat, and the Mol files for all compounds involved in the atom mappings are stored in MetaCyc-MOLfiles.tgz. The atom mapping encoding, as stored in atom-mappings.dat, is described at http://biocyc.org/PGDBConceptsGuide.shtml#node_sec_3.5. The atom mappings were computed by a technique described in (70). The error rate in computed atom mappings has been evaluated at <2%, although some atom mappings may possibly be incorrect due to specific enzyme activities that have not been taken into account.

In MetaCyc version 17.5, >10 100 reactions have computed atom mappings. The vast majority of reactions that lack atom-mapping data are either not completely mass balanced or they include substrates without an atomic structure. In a few rare cases, reactions were not processed because the computation would be too time consuming. In contrast, ∼5% of the reactions have multiple atom mappings, often due to symmetries in the compound structure. We tried to eliminate such duplicates, but we kept the cases where the enzyme might operate in more than one way.

The atom mappings are also used by the RouteSearch Tool (See Section below).

### New representation of glycan-degradation pathways

**Figure 1.**
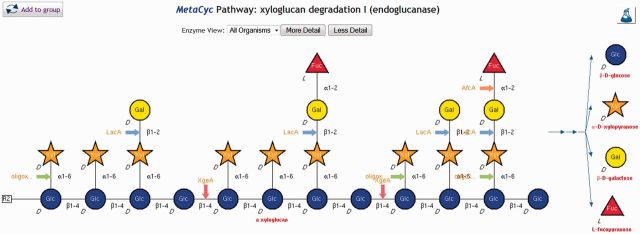
The new glycan degradation pathways use symbolic representation to illustrate the structures of complex glycan molecules. Colored arrows show the sites that are cleaved by enzymes and provide hyperlinks to those enzymes. The final products produced by the combined degradation of the polymer by all enzymes are listed on the right side of the diagram.

The degradation of large and complex glycan polymers poses a challenge for standard pathway diagrams. Rather than a linear process, the degradation of such polymers often consists of multiple types of enzymes simultaneously attacking different types of bonds within the polymer. The enzymes work in parallel, resulting in the liberation of small fragments. Because no particular order exists for these attacks, an attempt to show the process as linear, involving specific intermediates, is misleading. To overcome this limitation, we developed a new type of pathway diagram that shows the precise location of the sites attacked by the different enzymes by using color-coded arrows pointing to the cleaved bonds within the polymer structure (Figure 1). To make these diagrams easier to comprehend, we simplified the polymer structure by using glycan monomers as the basic building blocks. These pathways are often shown as a complex reaction, with the initial polymer structure on the left, and the final small products on the right.

Pathway Tools now supports the symbolic representation of glycans recommended by the Consortium for Functional Glycomics (CFG) and uses the Glyco-CT format for the import/export of such structures. To enable the curation of glycan structures, we developed a Pathway Tools interface for the GlycanBuilder software (71,72). GlycanBuilder is a tool that enables fast and intuitive drawing of glycan structures and was originally developed as the main interface for structure searches and results display in the EUROCarbDB databases.

The introduction of the symbolic structures did not replace the existing atomic structures—glycan molecules in MetaCyc may contain both the regular atomic structure that is used for all chemical compounds, and the CFG symbolic representation.

### Kinetic data in PGDBs

We have recently revised the types of enzyme kinetic data that can be captured in Pathway Tools PGDBs, the interface used to enter these data and the presentation of the data. When the reaction is reversible, capturing the optimal temperature and pH for each direction is now possible. K_m_, V_max_, K_cat_ and specific activity are now collected separately for each reactant, including for alternative substrates. K_i_ values for inhibitors are collected as before.

Version 17.5 of MetaCyc includes 3883 enzymatic reactions with Km data (5965 Km values), 242 enzymatic reactions with Vmax values, 390 enzymatic reactions with Kcat values and 201 enzymatic reactions with specific activity values.

**Figure 2.**
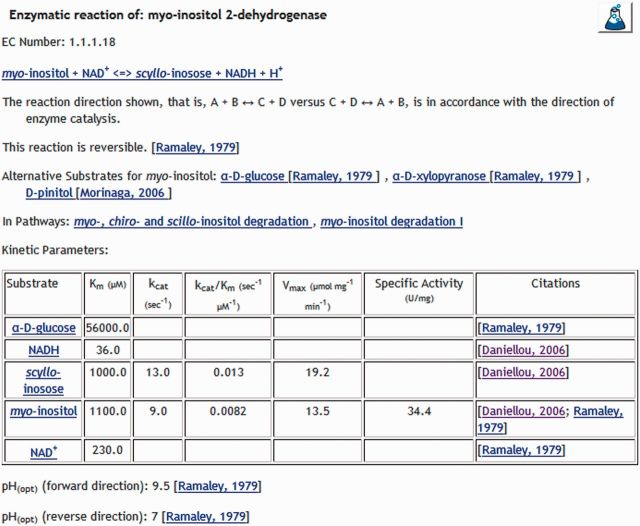
This figure illustrates some of the different types of enzymatic kinetic data that can be captured and presented by Pathway Tools. The software lets the curator enter the data using the units reported in a paper, and converts them automatically to the standard units. When possible, the catalytic efficiency is computed automatically and included in the table. Temperature and pH optima can be captured differently for the two directions of a reversible reaction.

In addition, all kinetic data are now presented in a table format that makes it much easier to read (Figure 2).

### New representation of the EC system in MetaCyc

**Figure 3.**
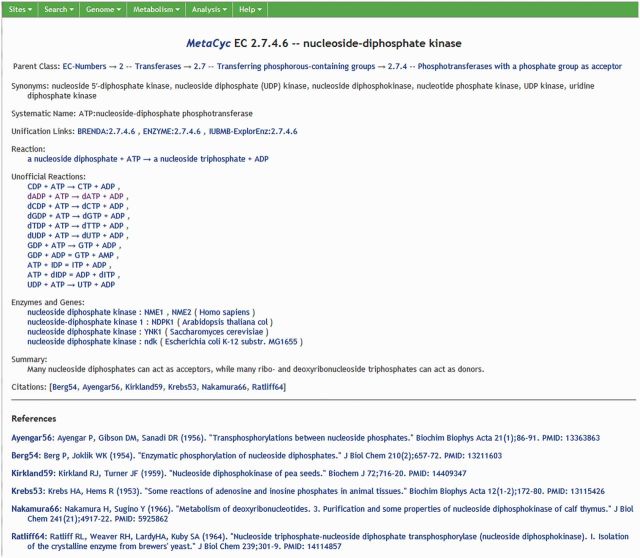
EC numbers are now database objects that have their own pages. An EC-Number page includes all of the information defined by the EC, and additional information that includes a list of unofficial reactions (see text for details) and a list of enzymes determined by the software to fit the definition of the EC number.

The Enzyme Commission (EC) classifies enzymes based on the reaction(s) that they catalyze (see http://www.chem.qmul.ac.uk/iubmb/enzyme/rules.html). Since its creation, Pathway Tools has encoded this information by assigning the EC number to the reaction catalyzed by the enzyme, with a limitation that only one EC number could be assigned to each reaction (although multiple reactions could be assigned to the same number). However, this approach gave limited compatibility with the many-to-many relationship between EC numbers and reactions that is used by the EC system. To increase compatibility with the EC system we have implemented a new way of encoding EC numbers. A new object type (EC-number) was added to the database to represent EC numbers (Figure 3). These EC-number objects have their own page, which contains the information drafted by the EC as well as links to several external databases. Any number of reactions can be linked to these EC-number objects, either as ‘official’ EC reactions, meaning that the reaction precisely matches the reaction(s) specified by the EC for this EC number, or as ‘unofficial’ EC reactions, meaning that while the reaction is not identical to the one used by the EC, it is implied to be catalyzed by this type of enzyme. When an enzyme has been assigned to all official reactions of a particular EC number, the software automatically recognizes that it fulfills the definition requirements for that EC number, and lists that enzyme in the EC-number page. Thus, we have implemented, in a dynamic computational manner, the principle of enzyme classification as defined by the EC, which is based on the reactions catalyzed by the enzyme.

### Data integration with other databases

#### EC classification

MetaCyc is regularly updated with data from the Nomenclature Committee of the International Union of Biochemistry and Molecular Biology (NC-IUBMB), which includes new and modified EC entries. The data are retrieved from the ExplorEnz database (www.enzyme-database.org) (73). The EC entries at ExplorEnz and MetaCyc are linked to each other.

#### NCBI taxonomy

The full NCBI Taxonomy database (74) is integrated into Pathway Tools, enabling specification of taxa using NCBI Taxonomy, and allowing taxonomic querying of MetaCyc pathways and enzymes. We continue to update the taxonomy entries with each major release of MetaCyc.

#### Gene ontology

The mapping between MetaCyc reactions and Gene Ontology (GO) process and function terms (75) is being continuously maintained by the GO Editorial Office at the EBI. An updated file is at http://www.geneontology.org/external2go/metacyc2go.

#### Links to other databases

During the past 2 years we added new links from MetaCyc to several external databases, which are listed in Table 3.

**Table 1.** List of species with >20 experimentally elucidated pathways represented in MetaCyc (meaning that there is experimental evidence for the occurrence of these pathways in the organism)

Bacteria		Eukarya		Archaea	
*Escherichia coli*	312	*Arabidopsis thaliana*	328	*Methanocaldococcus jannaschii*	25
*Pseudomonas aeruginosa*	70	*Homo sapiens*	229	*Methanosarcina barkeri*	21
*Bacillus subtilis*	60	*Saccharomyces cerevisiae*	172	*Sulfolobus solfataricus*	21
*Pseudomonas putida*	50	*Rattus norvegicus*	81		
*Salmonella typhimurium*	41	*Glycine max*	62		
*Pseudomonas fluorescens*	31	*Solanum lycopersicum*	55		
*Mycobacterium tuberculosis*	31	*Pisum sativum*	55		
*Klebsiella pneumoniae*	26	*Mus musculus*	51		
*Enterobacter aerogenes*	25	*Nicotiana tabacum*	46		
*Agrobacterium tumefaciens*	23	*Zea mays*	45		
		*Solanum tuberosum*	43		
		*Oryza sativa*	41		
		*Hordeum vulgare*	27		
		*Spinacia oleraca*	27		
		*Catharanthus roseus*	26		
		*Triticum aestivum*	25		
		*Bos taurus*	21		
		*Petunia x hybrida*	21		

The species are grouped by taxonomic domain and are ordered within each domain based on the number of pathways (number following species name) to which the given species was assigned.

**Table 2.** The distribution of pathways in MetaCyc based on the taxonomic classification of associated species

Bacteria		Eukarya		Archaea	
Proteobacteria	1042	Viridiplantae	916	Euryarchaeota	137
Firmicutes	319	Fungi	343	Crenarchaeota	42
Actinobacteria	280	Metazoa	312	Thaumarchaeota	1
Bacteroidetes/Chlorobi	62	Euglenozoa	28		
Cyanobacteria	65	Alveolata	16		
Deinococcus-Thermus	28	Amoebozoa	10		
Tenericutes	18	Stramenopiles	7		
Thermotogae	25	Fornicata	4		
Aquificae	16	Rhodophyta	4		
Spirochaetes	12	Haptophyceae	4		
Chlamydiae -Verrucomicrobia	7	Parabasalia	3		
Planctomycetes	6				
Chloroflexi	5				
Fusobacteria	4				
Nitrospirae	2				
Thermodesulfobacteria	2				
Chrysiogenetes	1				

For example, the statement ‘Tenericutes 18’ means that there is experimental evidence for at least 18 MetaCyc pathways for their occurrence in members of this taxonomic group. Major Taxonomic groups are grouped by domain and are ordered within each domain based on the number of pathways (number following taxon name) associated with the taxon. A pathway may be associated with multiple organisms.

**Table 3.** During the past 2 years we added new links from MetaCyc to the following external databases

Database name	Description	URL
Direct links		
dictyBase	A *Dictyostelium discoideum* model organism database	dictybase.org
DIP	A database of interacting proteins	dip.doe-mbi.ucla.edu
DisProt	A database of protein disorder	disprot.org
EuPathDB	A eukaryotic pathogen database	eupathdb.org
Expression atlas	A database of analyzed ArrayExpress Archive results	www.ebi.ac.uk/gxa
FlyBase	A *Drosophila melanogaster* model organism database	flybase.org
MINT	A molecular interaction database	mint.bio.uniroma2.it
PDB	A database of 3D structures of large biological molecules	rcsb.org/pdb
PDBsum	A pictorial database of PDB structures	www.ebi.ac.uk/pdbsum
PhosphoSitePlus	A database for protein post-translational modifications	Phosphosite.org
PRIDE	A proteomics identifications database	ebi.ac.uk/pride
Protein model portal	A database of protein models computed by comparative modeling methods	proteinmodelportal.org
Rhea	A manually annotated database of chemical reactions	ebi.ac.uk/rhea
STRING	A database of known and predicted protein-protein interactions	string-db.org
Swiss-model repository	A database of annotated three-dimensional comparative protein structure models generated by Swiss-Model	swissmodel.expasy.org/repository
‘In-family’ type links		
CAZy	A carbohydrate-active enzymes database	cazy.org
InterPro	A protein sequence functional analysis database	ebi.ac.uk/interpro
PANTHER	A database for protein analysis through evolutionary relationships	pantherdb.org
Pfam	A protein families database	pfam.sanger.ac.uk
PRINTS-S	A database of protein family fingerprints	bioinf.manchester.ac.uk/dbbrowser/sprint
ProDom	A database of protein domain families	prodom.prabi.fr
PROSITE	A database of protein domains, families and functional sites	prosite.expasy.org
SMART	A simple modular architecture research tool	smart.embl.de

## EXPANSION OF BIOCYC

The BioCyc databases are organized into three tiers.

- Tier 1 PGDBs have received at least 1 year of manual curation. Although some Tier 1 PGDBs (e.g. MetaCyc and EcoCyc) have received decades of manual curation and are updated continuously, others are less well curated and are still in need of significant curation.
- Tier 2 PGDBs have received moderate amounts of review (less than a year), and may or may not be updated on an ongoing basis.
- Tier 3 PGDBs were created computationally and received no subsequent manual review or updating.

During the past 2 years, the number of BioCyc PGDBs increased from 1129 (version 15.1) to 2988 (version 17.1). The Tier 1 PGDB YeastCyc (*S. cerevisiae*), which has been curated for many years by the saccharomyces genome database, is now hosted at BioCyc.org and has undergone significant curation in the past year. The number of pathways in YeastCyc has grown from 154 in December 2012 to 259 in October 2013. The curation of fungal pathways will be one of our priorities for the next few years.

The HumanCyc PGDB (*Homo sapiens*, curated by SRI), the AraCyc PGDB (*Arabidopsis thaliana*, curated by the Plant Metabolic Network) and the LeishCyc PGDB (*Leishmania major* strain *Friedlin*, curated by a team from the University of Melbourne) have been upgraded to Tier 1 status, bringing the total of Tier 1 PGDBs to six (along with EcoCyc, MetaCyc and YeastCyc). As of version 17.1, Tier 2 includes 35 PGDBs, and Tier 3 includes 2947 PGDBs. Some Tier 2 PGDBs were provided by groups outside SRI. The database authors are identified on the database summary page (Analysis → Summary Statistics).

### Inclusion of Human Microbiome Project genomes

As fully sequenced and annotated genomes become available from the Human Microbiome Project (http://www.hmpdacc.org/catalog/grid.php?dataset=genomic&project_status=Complete), they are integrated into the BioCyc collection. Version 17.5 includes 891 such genomes.

## SOFTWARE AND WEB SITE ENHANCEMENTS

The following sections describe significant enhancements to Pathway Tools (which powers the BioCyc Web site) during the past 2 years.

### Object-specific sidebar on BioCyc web pages

**Figure 4.**
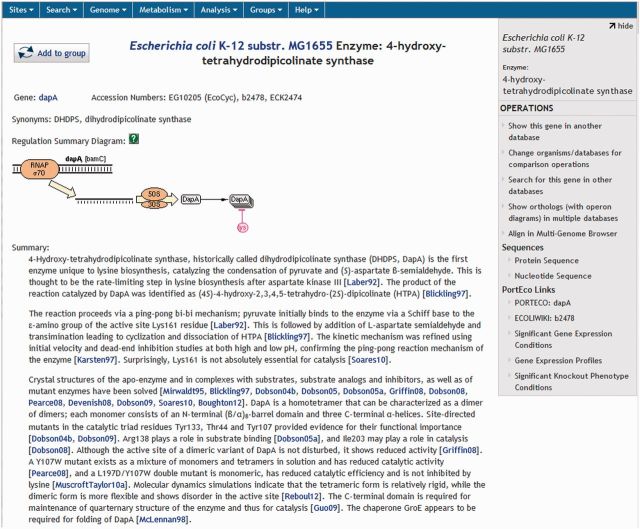
The new right-sidebar on BioCyc web pages contains operations that are specific to the currently displayed page. The operations and links available on the sidebar change depending on the type of object that is currently displayed. In this example, the operations and links are relevant to an *Escherichia coli* gene/protein page. Operations and links that are not specific to a particular object type are available from the menu bar at the top of the page and do not change.

A new right-sidebar appears on BioCyc web pages (Figure 4). This sidebar contains operations specific to the currently displayed BioCyc web page. For example, when a metabolic pathway page is displayed, the sidebar includes operations such as customizing the layout of the pathway and painting omics data on it. When a gene page is displayed, the sidebar includes operations such as displaying the gene sequence and producing a comparative genome browser view of that gene alongside specified orthologs.

### Improved compound structure graphics

The graphic display of the chemical structures on the compound and reaction pages has been re-implemented using the scalable vector graphics web standard, resulting in higher quality graphics. This improvement is currently visible only when using the recent versions of the Firefox and Chrome web browsers.

### Web Groups enhancements

A Web Group is a spreadsheet-like structure that can contain both Pathway Tools objects and other values such as numbers or strings. Like a spreadsheet, it is organized by rows and columns, and the user can add or delete any of them. A typical group contains a set of Pathway Tools objects in the first column (e.g. a set of genes generated by a search). The other columns contain properties of the object (e.g. the chromosomal position of each gene), or the result of a transformation (e.g. the reactions catalyzed by the gene products, or the corresponding genes from a different organism).

Web Groups can be created from search results, by importing data from external text files, and by adding objects individually from either their web pages or from another group. For example, a Web Group can contain a column of genes and columns of gene expression values, and the contents of the group can be painted onto a BioCyc metabolic map diagram using the Cellular Omics Viewer. Web Groups can be shared either publicly or with selected users.

Group transformations facilitate converting an existing group into a new group or into a new column in an existing group. Many new transformations have been added during the past 2 years, including several regulation-related transformations for genes (e.g. transforming a list of genes into a list of the transcription factors that regulate their expression), the ability to transform a group of genes into a group of the upstream promoters of those genes, to transform a protein into a list of regulatory DNA sites it binds to and to transform a compound into a list of proteins it is known to either bind to, activate or inhibit.

A relatively recent innovation is the ability to incorporate nucleotide and amino-acid sequence data as group objects. Such sequence data can be automatically added to groups that contain genes or proteins. Genes, promoters and transcription-factor binding sites can be transformed not only into their sequence but also into a list of their coordinates in the genome. A list of DNA regions or point locations (e.g. mutation locations) can be imported from a file to form a group, which could then be transformed into the set of genes nearest those regions.

The Web Groups interface also enables users to apply an enrichment/depletion analysis to the contents of a group (e.g. given a list of genes, the user can easily compute whether that list is statistically overrepresented for genes within specific metabolic pathways, or for genes that are regulated by particular transcriptional regulators).

### Metabolic RouteSearch

RouteSearch is a new web-based tool (accessible from the top menu command Metabolism → Metabolic Route Search) that generates reaction pathways connecting starting and ending metabolites specified by the user. Optional parameters include the number of routes to return, the maximum route length, the cost of using a native reaction (a reaction already found in the metabolic network of the organism, as opposed to a reaction that has to be imported from MetaCyc), the cost of losing an atom along the way and the atom species to take into account. Specifying the maximum amount of time allowed for searching for routes is also possible.

RouteSearch only returns linear pathways from the starting to the ending metabolite. Along that linear pathway, it computes the weighted sum of the atoms lost, based on the cost of atom loss provided by the user. To do so, it uses the atom-mapping data already computed for MetaCyc (see section Atom Mapping) because the atom mappings define which atoms are transferred between compounds. The objective of RouteSearch is to minimize this sum. RouteSearch also simultaneously minimizes the length of the pathways found, as the weighted sum of all reactions used to reach the ending metabolite adds to the overall cost.

Notice that searching for such optimal routes may not return well-known routes from a starting to an ending metabolite because the objective is to minimize both the number of reactions used and the number of atoms lost.

RouteSearch is a new tool and is still undergoing evaluation and potential redesign.

### Improvements to omics data display on the web cellular overview

The Cellular Omics Viewer enables users to paint an organism-specific metabolic map diagram for any BioCyc PGDB with multicolor highlighting to represent large-scale omics data sets, as well as to animate the highlighting to show temporal changes in the data. Any type of data that can be mapped to a compound, a reaction, an enzyme or a gene is supported, although the most common data types are gene-expression, reaction flux and metabolomics data. During the previous year, we reengineered the web-based Omics Viewer to improve its performance. Both speed and browser resource use were improved by one to two orders of magnitude. Currently the initial times to load data and display the resulting highlights are on the order of a few to the low tens of seconds.

#### Generating omics pop-ups

New functionality makes it possible to display per-node omics data in a pop-up window as a column chart, an x-y plot or a heat map. This new functionality is invoked by hovering the mouse over a reaction or metabolite of interest and selecting the ‘Omics’ option in the menu of the resulting pop-up window, which will graph the omics data for that object in column chart (bar) mode. Clicking different tabs converts the graph to an x-y plot or a heat map. Customizing the data labels is also possible.

**Figure 5.**
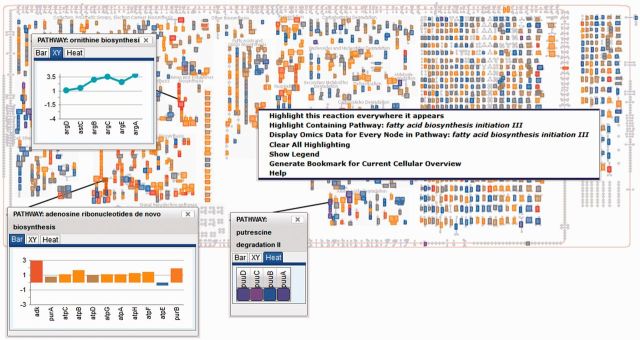
The Cellular Omics Viewer allows the user to paint omics data over the cellular Overview. New functionality enables the display of per-node omics data in a pop-up window as a column chart, an x-y plot or a heat map. The pop-ups can also be generated to show all the data for a given pathway. This figure also shows the pop-up that appears on right-clicking a reaction.

The same type of pop-up can be generated to show all the data for a given pathway. Right-clicking a reaction within the pathway opens a pop-up that includes the option ‘Display Omics Data for Every Node in Pathway’ (Figure 5).

#### Customizing a pathway diagram with omics data

**Figure 6.**
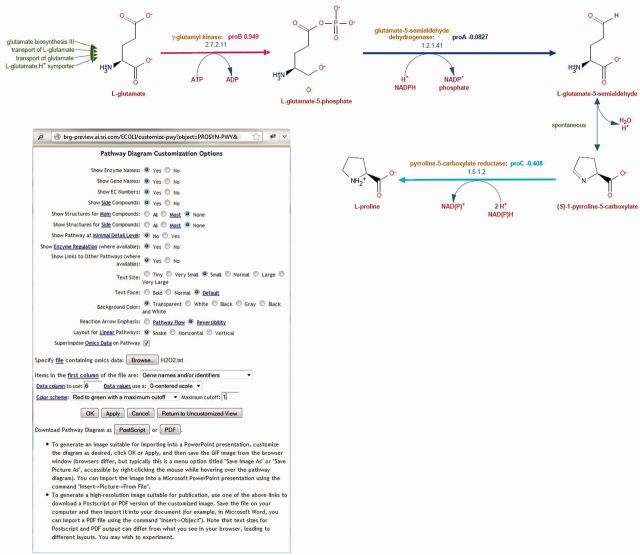
Pathway diagram customization is available via the web interface, and lets the user control many aspects of the pathway diagram. A new option allows painting user-supplied Omics data directly to the pathway. The modified diagram can be exported to a pdf or postscript format file for incorporation in presentations or manuscripts.

A new function enables the painting of omics data on a full-scale pathway diagram. The new functionality is invoked from the right-side bar by using the command ‘Customize Pathway Diagram’, which displays a window that includes an option for painting omics data onto the pathway diagram (Figure 6).

#### Generating a table of pathways with omics data

Generating a table displaying omics data painted onto small diagrams of all individual pathways is now possible. A ‘Show data’ selector has been added to the Omics Viewer dialog, which enables users to select whether they want the omics data painted on the cellular overview, the table of individual pathways or both.

### MetaFlux enhancements

The Flux Balance Analysis (FBA) module of Pathway Tools, called MetaFlux, enables the creation of steady-state quantitative metabolic flux models. MetaFlux is capable of solving FBA models, performing multiple gap-filling and performing multiple gene or reaction knockouts. Its latest enhancements include (i) the ability to specify compartments for the metabolites in the biomass reaction, the list of nutrients and the list of secreted metabolites; (ii) a much faster instantiation of generic reactions; (iii) some enhancements to the graphical user interface; and (iv) a new development mode called Fast-Gap.

In the regular development mode, gap-filling can be done simultaneously on reactions, nutrients, secretions and the biomass reaction. This regular development mode uses Mixed-Integer Linear Programming, which can be computationally time-consuming: it may require several hours of computing. The new Fast-Gap mode is limited to gap-filling only reactions, but it executes in a short time, typically in less than 1 min (at most a few minutes). Therefore, Fast-Gap can be used instead of the regular development mode when a fast answer is desired. Fast-Gap can also provide, in some cases, a more meaningful reaction gap-filling solution than the regular mode due to its use of a different optimization technique.

## ENHANCEMENTS TO THE DESKTOP VERSION OF PATHWAY TOOLS

The following enhancements only apply to the desktop version of the Pathway Tools software.

### Ability to save display state

The display state of Pathway Tools can now be saved to a file, which could be used for later restoration. Examples of display states that can be saved include the state of the omics viewers (including omics pop-ups), genome-browser tracks and cloned windows. The display-state file can be e-mailed to another user, who could then restore the exact same state on a different computer. Saving a display state to a file is invoked via the command **File → Save Display State to File**.

### Improved interface for the PGDB registry

Users that install Pathway Tools on their computer can download and install any of the PGDBs available on the BioCyc Web site by using an embedded utility called PGDB Registry (accessible from the command Tools → Browse PGDB Registry). This utility enables downloading and installing a PGDB with a few mouse clicks. However, the proliferation in the number of PGDBs available for download had resulted in a major slowdown of the utility. The interface of the utility has been completely redesigned, so that finding PGDBs within the registry is now fast.

## HOW TO LEARN MORE ABOUT METACYC AND BIOCYC

The BioCyc.org and MetaCyc.org Web sites provide several informational resources, including an online BioCyc guided tour (76), a guide to the BioCyc database collection (77), a guide for MetaCyc (78), a guide for EcoCyc (79), a guide to the concepts and science behind PGDBs (80) and instructional webinar videos that describe the usage of BioCyc and Pathway Tools (81). We routinely host workshops and tutorials (on site and at conferences) that provide training and in-depth discussion of our software for both beginning and advanced users. To stay informed about the most recent changes and enhancements to our software, please join the BioCyc mailing list at http://biocyc.org/subscribe.shtml. A list of our publications is available online (82).

## DATABASE AVAILABILITY

The MetaCyc and BioCyc databases are freely and openly available to all. See http://biocyc.org/download.shtml for download information. New versions of the downloadable data files and of the BioCyc and MetaCyc Web sites are released three or four times per year.

## FUNDING

National Institute of General Medical Sciences of the National Institutes of Health (NIH) [GM080746, GM077678, GM088849 and GM75742]; Department of Energy [DE-SC0004878 toward Bioenergy-related pathway curation]; National Science Foundation [IOS-1026003 and DBI-0640769 toward MetaCyc curation performed by the Plant Metabolic Network]. Funding for open access: NIH.

*Conflict of interest statement*. None declared.
